# Ductility Control via Nano-Precipitation at Grain Boundaries in Ti-Zr-Hf-Nb-Ta Multi-Principal Element Alloys

**DOI:** 10.3390/ma18071463

**Published:** 2025-03-25

**Authors:** Jiaying Li, Huibin Ke, Benpeng Wang, Liang Wang, Yunfei Xue

**Affiliations:** 1 School of Materials Science and Engineering, Beijing Institute of Technology, Beijing 100081, China; lijiaying0045@yeah.net (J.L.); wangbenpeng@bit.edu.cn (B.W.); lwangbit@bit.edu.cn (L.W.); xueyunfei@bit.edu.cn (Y.X.); 2 Yangtze Delta Region Academy of Beijing Institute of Technology, Jiaxing 314019, China; 3 National Key Laboratory of Science and Technology on Materials Under Shock and Impact, Beijing 100081, China

**Keywords:** multi-principal element alloys, precipitation, ductility, thermodynamics

## Abstract

The formation of nano-sized Hf_2_Fe precipitates at grain boundaries through Fe micro-alloying enhances the strength of Ti-Zr-Hf-Nb-Ta multi-principal element alloys (MPEAs), but this improvement comes at the cost of reduced ductility. Aging at 500 °C for just 30 min resulted in a marked reduction in elongation, from 17.5% to 7.5%. This decline is attributed to lattice mismatch between the precipitates and the matrix, as well as increased stacking stress at the grain boundaries. By adjusting the Fe composition and heat treatment parameters, the quantity of Hf_2_Fe at the grain boundaries of (TiZrHfNbTa)_100−x_Fe_x_ alloy was effectively controlled, achieving a balanced combination of strength of 1037 MPa and elongation of 14%. Furthermore, this method enabled ductility modulation over a wide range, with elongation varying from 2.65% to 19% while maintaining alloy strength between 955 and 1081 MPa, providing valuable insights for tailoring these alloys to diverse application requirements. The precipitation thermodynamics of the (TiZrHfNbTa)_100−x_Fe_x_ alloy was also investigated using the CALPHAD method, with thermodynamic calculations validated against experimental results, laying a foundation for more in-depth kinetic study of nano-size precipitates in these alloys. Additionally, the relationships between thermodynamics, precipitates evolution, and mechanical properties were discussed.

## 1. Introduction

Since their inception, multi-principal element alloys (MPEAs) have received extensive attention due to their design concept and excellent mechanical properties [1,2,3,4,5]. Unlike traditional alloys with a single principal element, MPEAs lack a clear principal element, and all atoms can be considered solute atoms. This unique characteristic promotes the formation of single saturated solid solutions, especially at high temperatures [1]. It was expected that such single saturated solid solutions would combine high mechanical strength, due to an enhanced solid solution strengthening (SSS), with high ductility, resulting from a homogeneous microstructure devoid of precipitate interfaces or brittle secondary phases [6,7,8,9]. Among these, Co-Cr-Fe-Mn-Ni MPEAs with a face-centered cubic (FCC) structure exhibit a remarkable balance of high strength and ductility. For example, the CoCrFeMnNi alloy achieves a yield strength of 759 MPa with elongation exceeding 70% [10,11]. Similarly, single-phase equiatomic CoCrFeNi alloys subjected to different heat treatments display yield strength ranging from 440 to 611 MPa, with elongation ranging from 38% to 50% [12,13,14]. On the other hand, Ti-Zr-Hf-Nb-Ta MPEAs with a body-centered cubic (BCC) structure also show excellent mechanical properties. He et al. [15] studied the mechanical properties of the TiZrHfNb, finding that it possesses both high strength (670 MPa) and high elongation (37%). Furthermore, an equiatomic TiZrHfNbTa alloy achieves an ultimate tensile strength of 974 MPa and maintains an elongation of 20% [16]. The unique design and mechanical properties of MPEAs, characterized by their high strength and ductility, have spurred extensive research into their potential applications [17,18,19].

While the formation of single saturated solid solutions contributes significantly to the properties of MPEAs, recent studies have also highlighted the pivotal role of precipitation strengthening in further enhancing their mechanical performance. Precipitation strengthening, which involves controlling the volume fraction, size, distribution, and characteristics of precipitates, offers a powerful means to improve strength [20,21]. Nevertheless, it often comes at the expense of ductility, presenting a challenge for alloy optimization. This compromise remains a major challenge for precipitation-strengthened alloys and is largely influenced by the size and characteristics of the precipitates [22,23,24,25,26]. For instance, the addition of Al to the CoCrFeNi MPEA matrix introduces the B2 phase. Rao et al. [27] demonstrated that increasing the Al content in Al_x_CoCrFeNi alloys (x = 0.3, 0.5, 0.7) raised the volume fraction of the B2 phase from 0.1% to 36%, resulting in a continuous increase in yield strength from 210 MPa to 600 MPa, albeit with a decrease in elongation from 97% to 8%. Similarly, Li et al. [28] studied the effects of Ni_3_Ti precipitates in CoCrFeNiTi_0_._3_ alloy prepared by in-situ laser powder bed fusion. The addition of Ti resulted in a network-like distribution of Ni_3_Ti precipitates, with a volume fraction of approximately 18%. This modification enhanced the yield strength from 509 MPa to 796 MPa, while the elongation dropped from 22.5% to 3.7%. In contrast, Tong et al. [29] mitigated the strength-ductility trade-off by refining the morphology and distribution of (Ni, Co)_3_Ti precipitates in CoCrFeNiTi_0.2_ alloys through tailored heat treatments, such as recrystallization and aging. Different from what was found by Li et al. [28], Tong’s results showed that compared with the parent CoCrFeNi alloy [14], the yield strength of the alloy increased from 440 MPa to 702 MPa, while elongation decreased only marginally, from 39% to 36%. This improvement was attributed to the formation of nanoscale precipitates (~4.1 nm) with a controlled volume fraction (~14.9%), which improved strength while minimally affecting elongation. These findings emphasize that precise adjustment of alloy composition and heat treatment can effectively modify precipitate size distribution and volume fraction, enabling a balance between strength and ductility in precipitation-strengthened MPEAs.

Building on these insights, Liu et al. [4] aimed to improve the yield strength of Ti-Zr-Hf-Nb-Ta MPEAs by adding Fe. They developed an MPEA with a BCC matrix and a small amount of Hf_2_Fe precipitates at the grain boundaries (referred to as HF1 alloy) and tested its mechanical properties. After aging at 500 °C for only 30 min, the yield strength of the alloy increased from 971 MPa to 1081 MPa due to the precipitation of Hf_2_Fe. However, the formation of precipitates at the grain boundaries also led to a significant reduction in elongation, from 17.5% to 7.5%.

In this work, the size distribution of Hf_2_Fe nano-precipitates at the grain boundaries of Ti_15_Zr_10_Hf_40−x_Nb_10_Ta_25_Fe_x_ (x = 3.35, 3.5) alloys was modified by altering Fe composition and heat treatment conditions. This approach enabled the achievement of a balance between strength and ductility, and also a series of alloys with a wide range of ductility. Additionally, the thermodynamics of these alloys were studied using the CALculation of PHAse Diagram (CALPHAD) to obtain reliable thermodynamic information, with computational results validated against experimental data. Finally, the evolution process of the precipitates and their impact on the mechanical properties of the alloys were discussed.

## 2. Materials and Methods

This work investigates a total of seven alloys, including two alloys as the HF1 alloy (Ti_15_Zr_10_Hf_36.5_Nb_10_Ta_25_Fe_3.5_) studied in previous work [4] and five newly designed alloys. One of the new alloys (HF1-600-30) retained the HF1 composition but underwent heat treatment at a different temperature to explore the effect of aging temperature on precipitate formation and mechanical properties. The remaining four alloys were designed with a modified composition (Ti_15_Zr_10_Hf_36.65_Nb_10_Ta_25_Fe_3.35_, denoted as HF2 alloy), reducing the Fe content by 0.15 at. % compared to HF1. These four alloys underwent different heat treatment processes to evaluate the effects of aging conditions on their microstructure and mechanical properties. The detailed heat treatment parameters for all alloys are provided in Table 1. The solution-treated alloys are named as HF1/HF2-ST; the aged alloys are named as HF1/HF2-aging temperature (°C)-aging time (min).

Alloys were produced by arc melting Ti, Zr, Hf, Nb, Ta, and Fe metals with high purity (>99.9 wt.%) in an argon atmosphere. After being repeatedly melted five times, the ingots were then drop-casted into a crucible with a dimension of Φ50 × 60 mm^3^. The alloy ingots were subjected to solution treatment at 1300 °C for 2 h and then water quenched. Subsequently, the solution-treated alloys were subjected to aging treatment at different temperatures and holding times and then water quenched again.

Scanning electron microscopy (SEM, ZEISS Gemini SEM 300, Beijing, China) equipped with a backscattered electron (BSE) detector was used to characterize the microstructure uniformity and grain boundary precipitates of alloys in Table 1. Samples for SEM observation were prepared using standard metallographic techniques and polished with silica suspension. Additionally, transmission electron microscopy (TEM, FEI Talos F200X, Beijing, China, with an accelerating voltage of 200 kV) was utilized to analyze the composition, crystal structure, size, and distribution of the grain boundary precipitates within the alloys. The TEM specimens were first mechanically ground to 100 µm and then ion-thinned until holes appeared.

Tensile specimens in a dog-bone shape with dimensions of 1 × 3 × 10 mm were machined from the samples by wire electrical discharge cutting. Uniaxial tensile tests with a fixed strain rate, 1 × 10^−3^ s^−1^, were conducted at room temperature using the CMT 4105 machine (Beijing, China). All the specimens at room temperature tensile tests were fractured. An extensometer was used to measure the strain during the tensile deformation. The tensile tests were repeated at least three times to ensure reproducibility of the results.

Thermodynamic calculations were performed using the TCHEA6, TCTI4, and TCNI12 databases in Thermo-Calc 2022b [30,31,32,33].

## 3. Results

### 3.1. Microstructural Characterization

**Figure 1:**
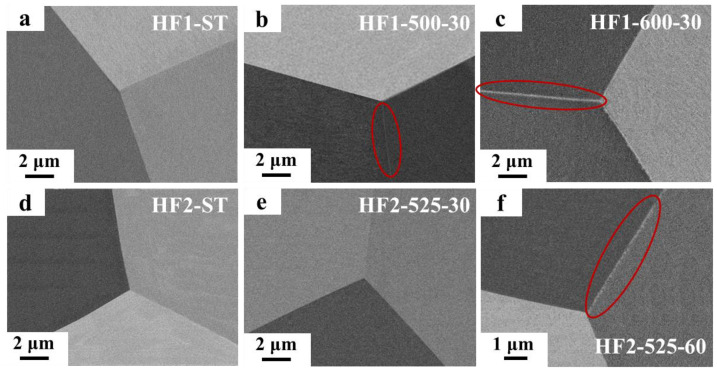
Microstructure of alloys in Table 1 observed by SEM: (**a**) HF1-ST alloy; (**b**) HF1-500-30 alloy; (**c**) HF1-600-30 alloy; (**d**) HF2-ST alloy; (**e**) HF2-525-30 alloy; (**f**) HF2-525-60 alloy.

**Figure 2:**
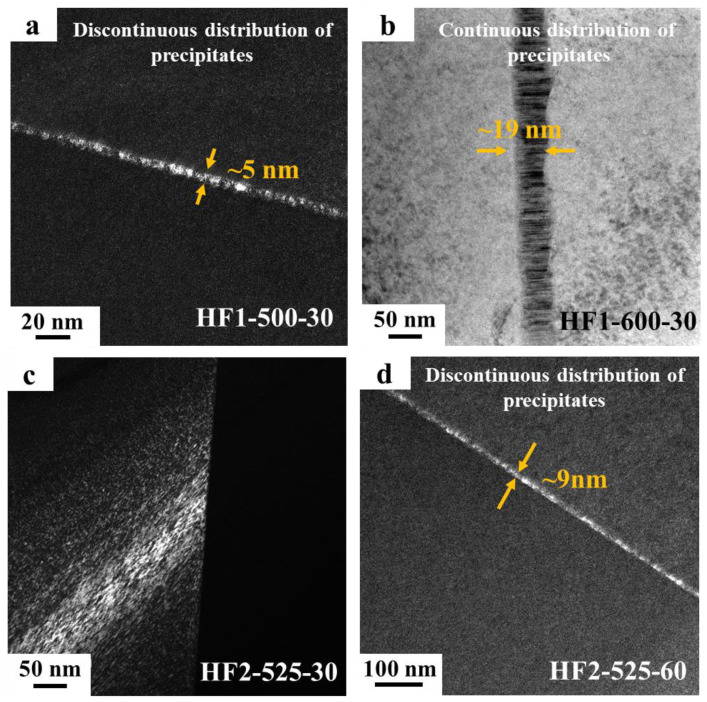
Distribution of precipitates at the grain boundaries of the alloys in Table 1 observed by TEM: (**a**) HF1-500-30 alloy; (**b**) HF1-600-30 alloy; (**c**) HF2-525-30 alloy; (**d**) HF2-525-60 alloy.

The alloys listed in Table 1 were characterized using SEM and TEM, with the results shown in Figure 1 and Figure 2, respectively. Figure 1a–c and Figure 2a–b display the microstructures of the HF1 alloys after different heat treatments. It can be observed that the HF1-ST alloy exhibited a single-phase structure without precipitates. In the HF1-500-30 alloy, SEM (Figure 1b) revealed very fine precipitates at the grain boundaries (within the red circle), and the TEM image (Figure 2a) showed that these precipitates were discontinuously distributed, with a thickness of approximately 5 nm. As the heat treatment temperature increased, the amount of precipitates in the HF1-600-30 alloy increased significantly, forming a completely continuous distribution at the grain boundaries, with a thickness of about 19 nm (Figure 2b). Figure 1d–f and Figure 2c–d display the characterization results of the HF2 alloys. Similar to the HF1-ST alloy, the HF2-ST alloy also showed no precipitates. In the HF2-525-30 alloy, no grain boundary precipitates were observed by either SEM (Figure 1e) or TEM (Figure 2c). However, in the HF2-525-60 alloy, precipitates appeared at the grain boundaries, with TEM revealing a discontinuous distribution and a thickness of approximately 9 nm (Figure 2d).

**Figure 3:**
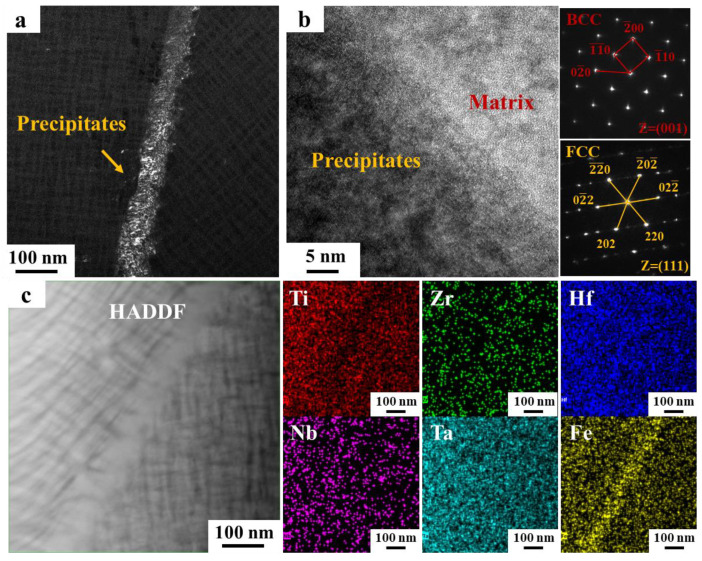
Microstructure of precipitates at grain boundaries in HF2-525-24h alloy: (**a**) Dark field image (DF) photograph of precipitates at grain boundaries; (**b**) High resolution transmission electron microscopy (HRTEM) images and corresponding fast Fourier transform (FFT) spectra at the interface between the precipitates and the matrix; (**c**) High angle annular dark field image (HAADF) of precipitates and corresponding EDS spectrum results.

To further investigate the elemental distribution and crystal structure of the precipitates in HF2 alloy, TEM was used to characterize the precipitates at the grain boundaries in the HF2-525-24h alloy, as shown in Figure 3. Figure 3a reveals continuous precipitates at the grain boundaries. High-resolution transmission electron microscopy (HRTEM) images, along with the corresponding fast Fourier transform (FFT), identified the matrix as BCC, while the precipitates at the grain boundaries were determined to be FCC, exhibiting a significant lattice mismatch with the matrix. Figure 3c displays a high-angle annular dark field (HAADF) image of the precipitates at the grain boundaries, along with the corresponding energy-dispersive spectroscopy (EDS) results. The surface scanning results reveal that the precipitates at the grain boundaries are enriched in Fe and depleted in Ti. This observation is consistent with Liu’s characterization of the HF1 alloy [4], which identified the precipitates as Hf_2_Fe with an FCC structure.

### 3.2. Mechanical Property

**Figure 4:**
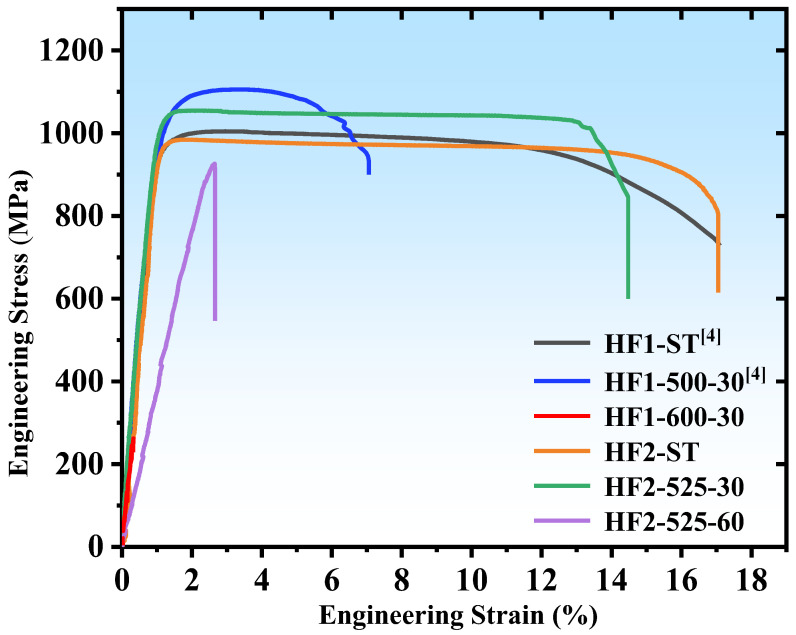
Tensile curves of alloys in Table 1 [4].

The mechanical properties of the alloys are shown in Figure 4 and Table 2. As shown in Figure 4, compared with the HF1-ST alloy, the strength of the HF1-500-30 alloy increased while the ductility decreased, which indicates that the formation of grain boundary precipitates enhances the strength of the alloy but reduces its ductility. The mechanical properties of the HF1-600-30 alloy reveal a significant drop in both strength and ductility compared to the HF1-500-30 alloy. A brittle fracture occurred before the yield point, with an average yield strength of about 271 MPa and no observable ductility. Comparing the properties of the HF2-ST alloy with the HF1-ST alloy, it is found that both the yield strength and elongation of HF2-ST (955 MPa, 19.0%) are close to those of HF1-ST (971 MPa, 17.5%). Compared to the HF2-ST alloy, it is found that the yield strength of the HF2-525-30 alloy increased to 1037 MPa, while retaining 14% elongation, achieving a favorable balance of strength and ductility. The HF2-525-60 alloy, in contrast, exhibits a strength of 960 MPa and elongation of 2.65%. In summary, by slightly modifying Fe composition (0.15 at.%) and aging temperature, a wide range of elongation from 2.65% to 19% could be obtained, and a balance of strength and ductility was obtained for HF2-525-30 alloy.

### 3.3. Thermodynamic Calculation

**Figure 5:**
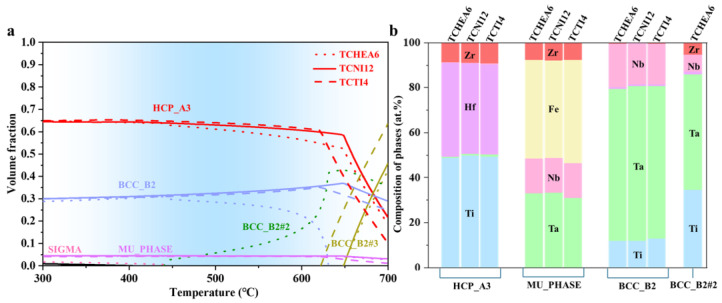
Thermodynamic calculations of precipitates in HF1 alloy by including all phases in the databases: (**a**) Equilibrium volume fraction of precipitates as a function of temperature; (**b**) Composition of phases at 500 °C.

CALPHAD calculations were performed to investigate the thermodynamics of the precipitates in HF alloys. The calculations were done with the TCHEA6, TCTI4, and TCNI12 databases using Thermo-Calc (TC) [30,31,32,33]. Initially, calculations were performed by including all possible phases from the databases. The results are shown in Figure 5. As can be seen from Figure 5a, all three databases produced similar outcomes, with the matrix identified as a HCP phase, and precipitates as *μ* phase and BCC phases. Figure 5b shows the composition of all phases at 500 °C. The matrix is enriched with Hf and Ti, and the *μ* phase is enriched with Fe and Ta. These results differ significantly from the experimental results, where the matrix was BCC, and the grain boundary precipitate phase was identified as Hf_2_Fe (FCC).

**Figure 6:**
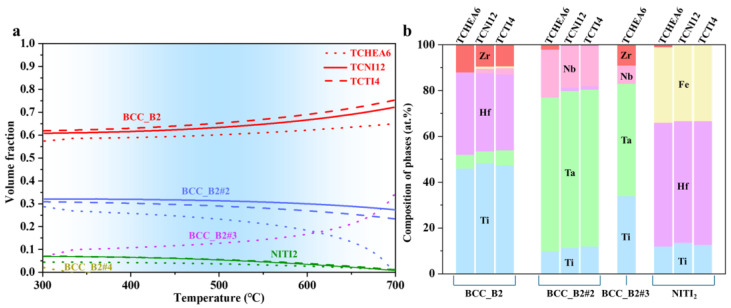
Thermodynamic calculations of precipitates in HF1 alloy by including the specified phases (BCC, FCC, NITI2) in the databases: (**a**) Equilibrium volume fraction of precipitates as a function of temperature; (**b**) Composition of phases at 500 °C.

To obtain accurate thermodynamic information for the system, the calculations were repeated by choosing only the BCC, FCC, and NiTi_2_ phases, based on experimental observations. The results are shown in Figure 6, with Figure 6a showing the equilibrium volume fraction of all phases as a function of temperature in the HF1 alloy and Figure 6b showing the composition of all phases at 500 °C. The calculation results from the three databases are similar, there are multiple BCC_B2 phases with different compositions and a NiTi_2_ phase enriched with Hf and Fe. Among them, the BCC_B2 phase is identified as the matrix phase, while the NiTi_2_ phase is the Hf-Fe precipitate observed by experiments. These findings are consistent with the experimental results. Within the temperature range shown in the diagram, the equilibrium volume fraction of the NiTi_2_ phase calculated by all three databases is less than 0.1. Besides the NiTi_2_ phase, a Ta-enriched precipitate (BCC_B2#2) was also obtained from the calculations. Thus, by selecting only the specified phases (BCC, FCC, NITI_2_), valid thermodynamic information for the HF alloy system can be obtained, providing a foundation for the development of kinetic models.

## 4. Discussion

### 4.1. The Influence of Nano-Sized Precipitates on the Mechanical Properties

The presence and characteristics of precipitates play a crucial role in determining the mechanical properties of MPEAs, particularly in terms of strength and ductility. In this study, precipitates were observed to significantly influence the mechanical properties of both HF1 and HF2 alloys, with a direct correlation between precipitate formation and strength enhancement, as well as a trade-off with ductility.

In the case of the HF1 alloy, precipitates formed at the grain boundaries upon aging at 500 °C for 30 min, leading to an increase in strength from 971 MPa to 1081 MPa. SEM and TEM analysis revealed that the grain boundary precipitates were very fine and discontinuously distributed, with a thickness of approximately 5 nm. This fine dispersion of precipitates obstructs dislocation motion, resulting in enhanced strength. However, as the aging temperature was increased to 600 °C (HF1-600-30), a significant increase in precipitate volume fraction occurred, leading to a completely continuous precipitate network at the grain boundaries with a thickness of 19 nm, which resulted in a marked reduction in ductility, with a brittle fracture occurring at around 271 MPa, significantly lower than that observed in the HF1-500-30 alloy. The effect of precipitate volume fraction on strength is well-documented, with the Orowan mechanism playing a key role in second-phase strengthening [34]. However, this comes at the cost of reduced ductility, as the precipitate network also serves as a stress concentrator, increasing the likelihood of crack initiation and propagation. This trade-off between strength and ductility is a common challenge in precipitation-strengthened alloys, particularly in high-strength alloys [35]. In the HF2 alloy, which has a 0.15 at. % lower Fe composition compared to HF1 alloy, no precipitates were observed in the solution-treated (HF2-ST) alloy, and the yield strength (955 MPa) was nearly identical to that of the HF1-ST alloy (971 MPa). Upon aging at 525 °C for 30 min (HF2-525-30), although no precipitates were visible through SEM or TEM (Figure 1e and Figure 2c), the yield strength increased to 1037 MPa while the elongation retained about 14%. This result suggests that fine nano-sized precipitates below the observation limit of TEM had formed at this condition. This subtle precipitation likely enhanced the alloy’s strength without causing a significant reduction in ductility. The increased elongation in the HF2-525-30 alloy compared to the HF1-500-30 alloy highlights the importance of controlling precipitate distribution. As the aging time increased to 60 min, discontinuous precipitates with a thickness of 9 nm were observed (Figure 2d). This led to a marked reduction in elongation, which dropped to 2.65% at a yield strength of 960 MPa.

The results from this study emphasize that precise control over the volume fraction of nano-sized precipitates can significantly affect the mechanical properties of MPEAs. As the volume fraction of nano-sized precipitates at the grain boundaries increases, the ductility of the alloys tends to decrease. In general, the volume fraction of the grain boundary precipitates formed in the four aged alloys follow this order from least to most: HF2-525-30 < HF1-500-30 < HF2-525-60 < HF1-600-30. This trend aligns with the ductility of the four alloys in Figure 4. During the early stage of grain boundary nano-precipitates formation, the ductility of the alloys drops while the strength of the alloys increases. However, as the precipitate volume fraction continues to increase, the alloy’s strength also declines. This phenomenon is consistent with observations in other alloys, such as the aging precipitation of stainless steel. Qin et al. [36] studied the aging precipitation of Mn18Cr18 austenitic stainless steel, where the primary precipitate, Cr_2_N, preferentially nucleates along the grain boundaries and grows into the matrix. As the aging time increased, the volume fraction of precipitates gradually increased, and the strength of the alloy remained relatively stable, while ductility deteriorated significantly. In our study, discontinuously distributed precipitates with a thickness of approximately 5 nm caused a reduction in ductility from 17.5% to 7.5%. In particular, the formation of nano-sized precipitates below the TEM detection threshold may play a crucial role in balancing strength and ductility. By slightly reducing the Fe composition (0.15 at.%) and increasing the aging temperature (25 °C), the HF2-525-30 alloy achieved an optimal balance between strength (~1037 MPa) and ductility (14%) compared to HF1-500-30 alloy.

### 4.2. Effect of Thermodynamics and Kinetics on Precipitation Evolution

The formation of precipitates is affected by both thermodynamic and kinetic factors. From a kinetic perspective, extending the aging time increases the volume fraction of precipitates, while raising the aging temperature accelerates the formation process, thus increasing the precipitate volume fraction. Thermodynamically, the alloy composition and heat treatment temperature both impact the precipitate formation. This effect can be described by the equilibrium solute product or the equilibrium volume fraction of the precipitates. The equilibrium solute product is defined as the product of the concentrations of the solute elements in the alloy matrix at equilibrium, each raised to the power of their respective stoichiometric coefficients in the precipitate phase. Mathematically, it can be expressed as:

(1) $$\bar{K_{sp}}=\prod_{i}{\bar{c}}_{i}^{x_{i}}$$

In the formula, $\bar{K_{sp}}$ is the equilibrium solute product of the precipitate, $\bar{c_{i}}$ represents the equilibrium composition of dissolved elements in the alloy matrix, while $x_{i}$ represents the composition of elements in the precipitate phase. For a given alloy, the equilibrium solute product is essentially a measure of the driving force of the precipitation. The higher the equilibrium solute product, the lower the driving force of the precipitates. For a given precipitate, the equilibrium product is only affected by temperature. It usually increases as temperature increases.

**Figure 7:**
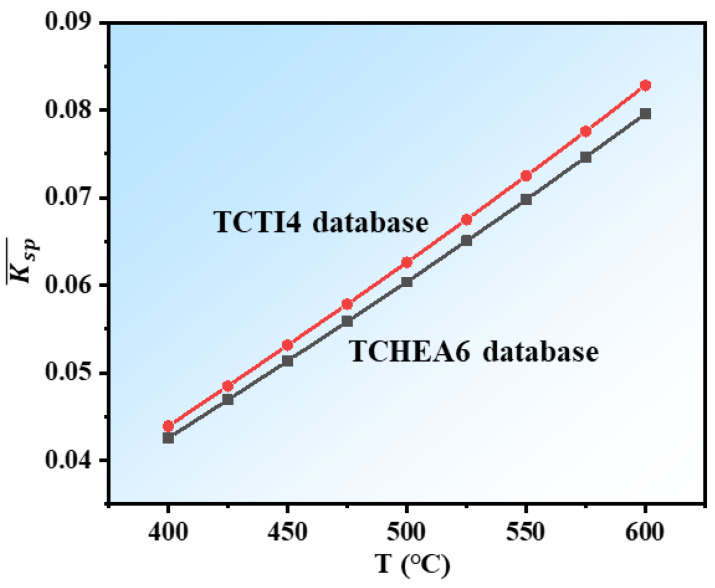
Equilibrium solute product of Hf_2_Fe precipitates as a function of temperature calculated by TCHEA6 and TCTI4 databases.

In this study, the curve for the NiTi_2_ phase in the alloy phase diagram calculated by TCNI12 and TCTI4 databases overlaps, so only the results from the TCHEA6 and TCTI4 databases are discussed below. The calculation results of the equilibrium solute product in the range of 400–600 °C of the TCHEA6 and TCTI4 databases are shown in Table 3 and Figure 7. For each temperature, the calculation results were averaged over ten different compositions of alloys (the content of Fe is in the range of 0.5–5 at.%). Therefore, these results are applicable to alloy compositions within this range. According to the calculation results, as the temperature increases, the equilibrium solute product gradually increases, indicating a decrease in the driving force for precipitation. Moreover, the equilibrium solute product at different temperatures of the TCTI4 database is slightly larger than the values calculated using the TCHEA6 database.

**Figure 8:**
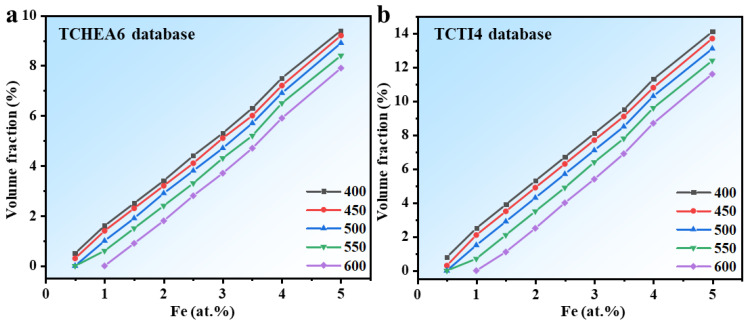
Equilibrium volume fraction of Hf_2_Fe precipitates as a function of Fe composition at different temperatures calculated by Thermo-Calc: (**a**) TCHEA6 database; (**b**) TCTI4 database.

Figure 8 shows the predicted equilibrium volume fraction of the Hf_2_Fe phase, calculated using the TCHEA6 and TCTI4 databases at different temperatures, as a function of Fe content. According to the calculation results, the phase equilibrium volume fractions obtained from the TCTI4 database are greater than those from the TCHEA6 database. It can also be observed that as the temperature increases or the Fe content decreases, the equilibrium volume fraction of the Hf_2_Fe phase decreases, making precipitation more difficult. Comparing Figure 1b,c and Figure 2a,b, it is evident that with constant alloy composition and aging time, an increase in aging temperature results in a larger amount and size of the precipitates. This is because the effect of aging temperature on the formation of precipitates is complex. Thermodynamically, it can reduce the driving force for precipitation, thereby decreasing the equilibrium volume fraction of the second phase. However, kinetically, higher temperatures can accelerate the precipitation process. In this case, kinetic effects dominate over thermodynamics, leading to an increase in the amount of the precipitates as temperature increases. Comparing Figure 1e,f and Figure 2c,d, it can be seen that with consistent alloy composition and aging temperature, a longer aging time results in larger sizes and greater amounts of the precipitates.

## 5. Conclusions

In this work, the size and distribution of the nano-precipitated Hf_2_Fe at the grain boundaries of the HF alloys were tailored by fine-tuning the alloy composition and modifying heat treatment parameters. Six alloys with varying Fe composition and heat treatments were prepared, with detailed characterization of precipitate distribution and mechanical property testing. Additionally, the thermodynamics of the alloy system were investigated via CAPHAD by using TCHEA6, TCTI4, and TCNI12 databases in Thermo-Calc. According to the results and discussions, the following conclusions were obtained:

(1) The size and distribution of nano-sized precipitates at the grain boundaries are sensitive to both alloy composition and heat treatment parameters and have a critical impact on the mechanical properties of MPEAs. By slightly adjusting the alloy composition (0.15 at.%) and varying the heat treatment parameters, a series of alloys with a wide range of elongation from 2.65% to 19% were developed, while maintaining alloy strengths between 955 and 1081 MPa, providing design flexibility for diverse applications.
(2) The formation of nano-sized precipitates below the TEM detection threshold plays a crucial role in balancing the strength and ductility. The HF2-525-30 alloy, in which no precipitates were observed in either SEM or TEM, exhibited a balanced combination of 1037 MPa strength and approximately 14% elongation.
(3) Thermodynamic calculations using Thermo-Calc, validated against experimental data, provided reliable phase stability information. By selecting specified phases (BCC, FCC, NITI_2_) within the TCHEA6, TCTI4, and TCNI12 databases, the equilibrium solute products of the Hf_2_Fe phase at 400–600 °C were obtained, along with the variation of its equilibrium volume fraction with Fe content at different temperatures.

## Figures and Tables

**Table 1:** The heat treatment process of studied alloys. The solution-treated alloys are named as HF1/HF2-ST; the aged alloys are named as HF1/HF2-aging temperature (°C)-aging time (min).

Alloy Name	Aging Temperature (°C)	Aging Time (min)
HF1-ST	——	——
HF1-500-30	500	30
HF1-600-30	600	60
HF2-ST	——	——
HF2-525-30	525	30
HF2-525-60	525	60
HF2-525-24h	525	24 h

**Table 2:** The summarized tensile properties of the alloys in Table 1.

Alloy Name	Yield Strength (MPa)	Elongation (%)	References
HF1-ST	971	17.5	[4]
HF1-500-30	1081	7.5	[4]
HF1-600-30	271 ± 6	0.35 ± 0.15	This work
HF2-ST	955 ± 25	19 ± 2	This work
HF2-525-30	1037 ± 17	14 ± 1	This work
HF2-525-60	960 ± 50	2.65 ± 0.15	This work

**Table 3:** Equilibrium solute product of Hf_2_Fe calculated by TCHEA6 and TCTI4 databases at different temperatures.

T/°C	Equilibrium Solute Product			
	TCHEA6 Database		TCTI4 Database	
	Average Value/10^−2^	Variance/10^−6^	Average Value/10^−2^	Variance/10^−6^
400	4.25	0.95	4.39	1.00
425	4.69	1.22	4.85	1.26
450	5.13	1.58	5.32	1.60
475	5.58	1.90	5.78	2.03
500	6.04	2.38	6.26	2.49
525	6.51	2.91	6.75	3.00
550	6.98	3.45	7.25	3.49
575	7.46	4.08	7.76	4.07
600	7.96	4.68	8.28	4.67

## Data Availability

The original contributions presented in this study are included in the article. Further inquiries can be directed to the corresponding author.
